# Challenges and opportunities for eliminating tuberculosis – leveraging political momentum of the UN high-level meeting on tuberculosis

**DOI:** 10.1186/s12889-019-6399-8

**Published:** 2019-01-16

**Authors:** Haruka Sakamoto, Sangnim Lee, Aya Ishizuka, Eiji Hinoshita, Hiroyuki Hori, Nanao Ishibashi, Kenichi Komada, Masataro Norizuki, Yasushi Katsuma, Hidechika Akashi, Kenji Shibuya

**Affiliations:** 1 Department of Global Health Policy, Graduate School of Medicine, Medical Building No.3, Hongo Campus, University of Tokyo, 7-3-1, Hongo, Bunkyo-ku, Tokyo, Japan; 2 Bureau of International Health Cooperation, National Center for Global Health and Medicine, Institute for Global Health Policy Research, Tokyo, Japan; 3 School of Global Studies and Collaboration, Aoyama Gakuin University, Kanagawa, Japan; 4 Bureau of International Health Cooperation, National Center for Global Health and Medicine, Tokyo, Japan; 5 Ministry of Health, Labour and Welfare of Japan, Tokyo, Japan; 6 Graduate School of Asia-Pacific Studies, Waseda University, Tokyo, Japan

**Keywords:** Tuberculosis, Global Health, Health policy, Global Fund, Stop TB partnership, United nation high-level meeting

## Abstract

**Background:**

As demonstrated by the United Nations High-Level Meeting on tuberculosis (TB) held in September 2018, the political momentum for TB has been increasing. The aim of this study was to analyze the current challenges and opportunities for global TB control and, with specific focus on policies surrounding TB control, to reveal what kinds of efforts are needed to accelerate global TB control.

**Methods:**

We organized two expert meetings with the purposes of assessing the current situation and analyzing challenges regarding TB control. By applying Shiffman and Smith’s framework which contains four categories; Actor, Ideas, Political context, and Issue characteristics, we analyzed the challenges and opportunities for global TB control based on the findings from the two expert meetings.

**Results:**

In the Actor Category, we found that although there has already been active engagement by non-governmental organizations (NGOs), civil society organizations (CSOs) and private sectors, there still remained an area with room for improvement. In particular, the complexities behind varying drug regulatory and procurement systems per country hindered the active participation of the private sector in this area. As for the Ideas category, due to an increasing threat of antimicrobial resistance and growing number of global migrations, TB is now widely recognized as a health security issue rather than a purely health issue. This makes TB an easier target for political attention. As for the Political category, having the UN High-Level Meeting itself is not enough; such meetings must be followed up by actual commitments from heads of states. Lastly the issue characteristic indicates that the amount of funding for R&D for new drugs, vaccines and diagnostics for TB is not at an adequate level, and investment in childhood TB and missing cases are particularly in need.

**Conclusions:**

This study provides important insight into the current status of global efforts toward end TB epidemic. The outcomes from the UN high-level meeting on TB need to be closely monitored will be crucial for the progress towards this goal.

## Background

Tuberculosis (TB) is the leading cause of death from communicable diseases worldwide; every year, 10.0 million people develop TB and 1.6 million people die from the disease [1]. TB is listed as a major health challenge in the Sustainable Development Goals (SDGs) as stated in Goal 3.3: “by 2030, end the epidemics of HIV/AIDS, TB, Malaria and neglected tropical diseases and combat hepatitis, water-borne diseases and other communicable diseases.” The first global ministerial conference on ending TB in the sustainable development era was held in November 2017, and the “Moscow Declaration to End TB” was adopted. Most recently, in September 2018, the first United Nations high-level meeting on TB was held. There is clearly a strong political momentum at the global level for ending the TB epidemic by 2030.

Despite significant progress in the past decades, at the current rate of progress, the SDG target on TB will not be achievable [2]. Currently, the annual rate of decline in TB incidence is around 1–2% while the rate would need to be 4–5% by 2020 and over 10% by 2025 in order to achieve the goal of ending the epidemic by 2030 [3]. Moreover, new challenges are emerging, including an increase of multi-drug resistant tuberculosis (MDR-TB), a large number of missing cases (the 6.4 million cases reported represented 64% of the estimated 10.0 million new case that occurred in 2017), and global migration, which will impose a huge financial and political burden on the TB control [4]. In this paper, we analyze the current status and challenges for TB control with specific focus on policies surrounding TB control so as to reveal what kinds of efforts are needed to accelerate global TB control.

## Methods

Several analytical frameworks have been developed in global health policy analysis including those proposed by Shiffman (2007), Shiffman and Smith (2007), Clark (2000) and McAdam (1990) [5–8]. In this study, we applied Shiffman and Smith’s framework which contains four categories; actor, ideas, political context, and issue characteristics. The definitions of each category are summarized in Table 1.

**Table 1:** Shiffman and Smith’s framework

Category	Factor	Explanation
Actor	1. policy community cohesion	The degree of cohesion among stakeholders
	2. leadership	The presence of individuals capable of raising awareness and mobilizing resources, and are widely acknowledged by the community
	3. guiding institutions	The effectiveness of organizations or coordinating mechanisms with a mandate to lead the issue
	4. civil society mobilization	The extent to which grassroots organizations have mobilized the necessary support from international and national political authorities
Idea	5. internal frame	The way policy community agree on the definition of, causes of, and solutions to the issue
	6. external frame	The way to portray the issue outside of the policy community
Policy environment	7. policy window	Political momentum when global community favorably align the issue
	8. global governance structure	The degree to which norms and institutions operating in a sector provide a platform for effective collective action
Issue characteristics	9. credible indicators	Clear and measurable indicators which can trace the severity and progress
	10. severity	The size of the burden relative to other issues (i.e., mortality)
	11. effective interventions	The extent to which proposed means of addressing the problem are clearly explained, cost-effective, evidence-based, easy to implement and affordable

Authors from the Institute for Global Health Policy Research (iGHP), National Center for Global Health and Medicine (NCGM) and the Department of Global Health Policy (GHP), University of Tokyo organized two expert meetings. The purposes of these meetings were to assess the current situation and analyze challenges regarding prevention and control of TB, and to identify types of efforts needed to accelerate global TB control.

The first meeting was held in April 2018 and was attended by a wide range of experts including officials from the Japanese government, Japan International Cooperation Agency (JICA), NCGM, Japan Anti-Tuberculosis Association (JATA), the Global Fund to Fight AIDS, Tuberculosis and Malaria (the Global Fund) and others. The second meeting was held in May 2018 and open to the public. In addition to the organizations above, the meeting was attended by representatives from non-governmental organizations (NGOs), civil society organizations (CSOs), and the private sector (mainly pharmaceutical and medical device companies). Based on the discussion at the two meetings, we categorized main findings into four categories according to Shiffman and Smith’s framework.

### Patient and public involvement

Patients and members of the public were not directly involved in this study. This study is a policy analysis and does not focus on any individuals nor general public.

## Findings

Seventy-seven people attended the meetings; 8 (10%) were public officials, 18 (23%) were from the private sector, 17 (22%) were from NGOs/CSOs, 13 (17%) and 11 (15%) were from academia and development partners, respectively (the remaining 10 (13%) were from other sectors). The outcome of the meetings is summarized in Table 2, according to the Shiffman and Smith’s framework.

**Table 2:** challenges and opportunities for tuberculosis

Category	Challenges	Opportunities
Actor	- The private sector has not been effectively involved in the control and treatment of TB.	- Three key organizations – WHO, the GF and the Stop TB partnership have been enhancing the policy community.
	- Most NGOs are facing a lack of financing, which may weaken their capacity for implementation.	
		- Active engagement of NGOs, CSOs and community organizations.
Ideas	- Need to pay more attention to human-rights aspects of TB prevention and treatment.	- Clear and well-established models of the causes of TB and of interventions for reducing the TB burden.
		- Well framed as security issues.
Political context	- Given the increased number of health issues highlighted at UN high-level meetings in recent years, it is uncertain how much impact such a meeting on TB has on the attention and priorities of the high-level leaders.	- Already listed in MDGs and SDGs
		- Ministerial conference on ending TB in the sustainable development era in Moscow in 2017
		- UN high-level meeting on TB in 2018
Issue characteristics	- R&D for new drugs, vaccines and diagnosis are too slow and funding is limited.	- Top infectious killer globally
		- Substantially affects children
		- Well-known interventions (4-regimen, 6-months)

### Actor power

Over the past decades, WHO, together with the Global Fund and the Stop TB partnership (STBP) have been the guiding institutions in the fight against TB. By publishing several guidelines and action plans including “the End TB Strategy” and “the Global Plan to End TB 2016 – 2020”, these organizations have contributed to enhancing policy community cohesion. Recently, WHO, the Global Fund and STBP launched a joint initiative titled “FIND. TREAT. ALL. #ENDTB,” to scale up prevention and control of TB so as to secure adequate care for millions of TB patients.

In addition to these efforts made by the WHO, the Global Fund and the STBP, experts at the two meetings emphasized the importance of contributions by community health workers, NGOs and the private sector. For example, community health workers including public health nurses played a significant role in the successful efforts to reduce the TB burden in Japan. Public health nurses, who were trained in patient-centered and human-rights based TB control programmes, delivered high quality care not only at the health facility level but also through a reach out to schools, work places and other community-based facilities. Their contribution to the disease detection and management resulted in the rapid decline in the TB prevalence in Japan. What was once 700/100,000 in 1950s, the prevalence rate declined annually by 10% between 1965 and 1978 (which is almost equal to the rate required to reach the global target by 2030) [9, 10].

Another important actor is the private sector, which contributed to TB control by developing new vaccines, treatment and diagnostic tools, in particular for MDR-TB, and also devised public-private partnership. The Global Health Innovation Technology Fund (GHIT Fund) founded in 2012 by the Government of Japan together with pharmaceutical companies, the Bill and Melinda Gates Foundation, and the Wellcome Trust (joined in 2015), is one such example. GHIT Fund aims to promote the development of new medical products and support innovation that addresses the needs of developing countries. As of March 2018, GHIT Fund has invested USD 190 million in 74 partnership projects (12.5% of the fund was allocated to TB), in which the organization aims to address market failures and incentivize research and development (R&D).

The actor category also refers to civil society mobilization. A large number of NGOs, such as Médecins Sans Frontières (MSF), RESULTS and community organizations have been actively participating in grassroots activities. As of May 2018, about 80% of partners of STBP are NGOs and CSOs, which indicates an active participation of these organizations in the fight against TB.

### Ideas

There are clear and well-established understanding of the causes of TB and models of interventions for reducing the TB burden. TB is caused by *Mycobacterium tuberculosis* and the standard treatment includes four antimicrobial drugs during a period of six months. The clear understanding of the cause and the treatment of this disease makes it easy for relevant stakeholders to unite around a common issue and potential solutions for it.

The emergence of MDR-TB has successfully framed the disease as not only a health issue, but also a security issue. More than 30% of antimicrobial resistance (AMR) related deaths occur among MDR-TB patients [11]. With a global momentum for combating AMR on the rise, framing TB as AMR-related security issue could well-position TB for capturing political attention, especially from heads of states, foreign ministers, finance ministers, and trade and economy ministers.

Another issue that makes TB a health security concern is the increasing prevalence of TB among immigrants and migrants. For example, in Japan, which is an intermediate TB burden country, foreign-born TB cases increased from 842 in 2007 to 1101 cases in 2014, with the most prominent rise occurring among those aged 20–29 years from 21.2% in 2007 to 44.1% in 2014 [12, 13]. This trend can be seen in many developed countries [14], and together with general concerns of increasing transnational movement of people, the idea of TB as a health security issue has now become widely recognized.

### Political context

There is already a political support for the fight against TB: both MDGs and SDGs listed TB as priority. Moreover, the year of 2018 was a historical year in the combat against TB. At the 71st United Nations General Assembly in 2016, member states unanimously adopted the resolution A/RES/71/159 entitled “Global health and foreign policy: health employment and economic growth”, in which it was decided to have a UN High-Level Meeting on TB in 2018. Following this resolution, the global ministerial conference on ending TB in the sustainable development era was held in Moscow in 2017. At the conference, health ministers adopted the “Moscow Declaration to End TB,” which now constitutes the basis of the efforts to end the TB epidemic. In September of 2018 at the UN General Assembly, the heads of States adopted the “Political declaration of the high-level meeting of the General Assembly on the fight against tuberculosis,” which called for a united and urgent global response to fight the epidemic and outlined concrete actions for increased investments and innovation. With the endorsement from the highest level of authorities by UN member states, the global commitment to fight the epidemic has never been higher.

### Issue characteristic

As for severity of the disease, deaths due to TB now exceed that of HIV/AIDS, and TB is now the top infectious killer globally [4]. Moreover, TB affects children, and the issue of childhood TB has been raised among the international stakeholders, and described in the “Roadmap for Childhood Tuberculosis” report [15]. In 2016, less than half (43%) of the estimated 1 million children with TB were reported to national TB programmes, indicating that there is a large number of undiagnosed and insufficiently treated children [4]. As a result, 253,000 children died of TB. Unrecorded cases are also of concern among adults. The 6.4 million cases reported represented 64% of the estimated 10.0 million new case that occurred in 2017, indicating that there are many TB patients who have no access to health care facilities or proper diagnosing and treatment.

As for effective interventions, the standard treatment is to provide a six-months course of four antimicrobial drugs. In addition, a great progress has been made in the development of new TB drugs in recent years. We now have a three-months, once-weekly regimen for TB prevention (isoniazid and rifapentine), and a nine-months regimen that cure 80% of MDR-TB [16]. There are two additional drugs – bedaquiline and delamanid – which have been approved by regulatory authorities [16]. However, the pace for developing new drugs, vaccines and diagnostics is slow and fundamental changes in R&D are needed. BCG vaccine was first initiated for more than 7 decades ago. Though this vaccine has been widely used globally, there is no other new vaccine developed and more effective vaccine is needed. The WHO *Global Tuberculosis Report* estimates that during the 2016–2020 period, two billion USD per year is needed for global TB R&D, while there was only a maximum of 0.7 billion USD available each year during 2005–2015 [4].

Another important aspect of TB is the huge financial burden it places on patients and their families. In addition to treatment costs, TB patients are required to take a leave of absence from work leading to risk of impoverishment [17–19]. Tanimura et al. reported in 2014 that the total cost of TB treatment was equivalent to 58 and 39% of reported annual individual and household income, respectively [20]. In the midst of increasing momentum toward Universal Health Coverage (UHC), providing financial risk protection for TB patients and their families is now of great concern.

## Discussion

With regard to the actor category, key three organizations – the WHO, the GF and the STBP – have played important roles as guiding institutions in increasing cohesion around TB targets in the policy community. The private sector, NGOs and CSOs have played significant roles as well. However, challenges remain especially for the engagement of the private sector. Even if several private companies have joined the STBP, they constitute only around 7% of the STBP membership and a platform is lacking for the private sector to actively engage in the prevention and treatment of TB.

During the meetings that we organized in May 2018, several private companies emphasized the complexities behind varying drug regulatory and procurement systems per country that resulted in difficulties of introducing new medicines and tools for diagnosis in TB endemic countries. As the private sector plays an important role in promoting R&D on new drugs, vaccines and tools for diagnosis, it is crucial to create an environment that can maximize the benefits from private sector engagement. This would include adequate financing of R&D, simplified procurement systems by the WHO as well as in each country, and a global framework on drug regulatory and approval.

Civil society engagement is also a key to continuing and further scaling up of interventions on TB. Several NGOs and CSOs expressed strong concerns regarding inadequate financing for activities at the grassroots level. According to the WHO *Global Tuberculosis Report*, 9.2 billion USD per year is required for the implementation of TB interventions, while only 6.9 billion USD was available for countries to use in 2016, leaving a funding gap of 2.3 billion USD [4]. It is thus important to raise financial support for the fight against TB, and allocate these funds adequately into implementation activities at the grassroots level.

Regarding the idea category, participants of the meetings emphasized that TB should be regarded not only as an infectious disease but also as a health security issue, especially because TB is the only major drug resistant epidemic that is airborne. Moreover, a global increase in migration and immigration accelerates the spread of TB. The Ebola disease outbreak was perceived as a health security issue and caught significant political attention [21]. It would be a feasible strategy to also emphasize the security aspect of TB. In addition, unlike HIV/AIDS, TB is lacking the framework and recognition as a human rights issue [22]. TB requires long treatments and sometimes even isolation of patients, which can lead to stigmatization [23]. Such a stigmatization can delay or hinder the patients treatment seeking behavior. To overcome such social barriers, inclusion of human rights-based and patient centered approaches in framing of the disease would also be needed.

As for the political context, it is certain that TB now gets attention at the highest political level more than ever. Other health challenges such as HIV/AIDS, non-communicable diseases (NCDs), and AMR, caught attention from higher political level after being subject to UN high-level meetings and ministerial conferences. The UN High-Level Meeting on HIV/AIDS in 2001 was the first-ever high-level meeting dedicated to a health issue. Since then, health issues have appeared more frequently on the agenda of high-level meetings; HIV/AIDS and AMR in 2016, TB and NCDs in 2018, and UHC in 2019. Given the increasing number of health issues highlighted in recent years, it is uncertain how much impact they have on the attention and priorities of the high-level leaders. UN high-level meeting on TB just finished in September 2018, and further monitoring is needed to ensure adequate political attention and financial resources for TB.

Moreover, just holding UN high-level meetings on TB is not enough; such meetings must be followed by actual commitments from heads of states. The fundamental challenges to TB entail the lack of financing. Being in the position to greatly influence financial priorities, it is important for the heads of states from low- and middle-income countries with moderate to high TB burden to put TB high on their country’s agenda and increase domestic financing on TB control. In particular, countries that are transitioning away from Global Fund’s support need to devise strategies for continued efforts using domestic financing. Heads of states from high- income countries need to raise additional financial support for TB control as well, including the contribution to the 6th replenishment of the Global Fund and to the Global Drug Facility (GDF) operated by the STBP.

Lastly with regard to issue characteristics, as pointed out during the meetings, childhood TB is now of great concern. When Japan successfully reduced the TB incidence between 1965 and 1978, the overall rate of incidence decline was 10%, while it was 15–30% among children [24]. The rate of decline in incidence for childhood TB is typically faster than the overall rate of decline. This metric could be used as an indicator for successful TB control. The global community is encouraged to set specific target for childhood TB. Moreover, child-friendly diagnostics and treatments are urgently needed. Although there are several new diagnostics and drugs that have been discovered in recent years, further efforts are needed for developing shorter and more simple drug regimens, especially for children. Experts from our meetings emphasized the importance and uniqueness of GHIT Fund activities and underscored the need for this kind of innovative public-private partnerships with a specific focus on R&D.

As to financial burden due to TB, Japan’s experience would be of value for other countries. In Japan, universal health insurance was introduced in 1961 and public subsidy for TB patients (starting in 1951) played important roles for controlling the financial burden of TB patients. With the financial protection offered by health insurances, patients accessed health care facilities for diagnosis, and their treatment cost was fully funded by the government once they were diagnosed with TB [25]. Public subsidy also provided incentives to private health care facilities to enter TB control programs and contributed to enforce registration and standardization of TB treatment. This mixture of financial risk protection –universal insurance and public subsidy was the key driving force for Japan to reduce TB burden and to attain UHC. Research has shown that the majority of patients stopped their TB treatment before 6 months due to financial reasons [26–28]; making systems for covering treatment costs important. Currently, the Global Fund supports the provision of TB diagnosis and treatment for free (similar to public subsidy in Japan), while most of the countries are now on the way toward creating universal health insurance. This good mixture – public subsidy and health insurance – are keys for success for TB control as well as attaining UHC.

Another remaining issue in the era of the SDGs is to address the missing cases and TB patients in vulnerable populations– “the last one mile”. As the population constituting “the last one mile” differs across countries, each country needs to identify its vulnerable populations. Addressing “the last one mile” requires a comprehensive set of efforts including adequate medicines and diagnostic tools as well as accessible and accurate information.

### Limitations of this study

As we only focused on challenges and opportunities for TB control raised during the two expert meetings, this manuscript does not cover all the challenges TB control faces. For example, latent tuberculosis infection (LTBI) is now of great concern, especially in aging population, but we did not discuss anything about LTBI in this manuscript. In addition, majority of participants from the two expert meetings are Japanese. Although Japan is an intermediate burden country, there was no experts attending from low- or middle- income countries nor TB high-burden countries. Further researches are needed with more diverse stakeholders.

## Conclusion

In 2018, 136 years have passed since the discovery of *Mycobacterium Tuberculosis* by Robert Koch as well as 25 years since the WHO declared TB as a global health emergency. Significant progress has been made in the fight against the disease, especially since the creation of the Global Fund and the STBP. However, several challenges remain. This year is a historical year for the prevention and control of TB and is crucial for attaining the goal of ending the epidemic by 2030.

## Abbreviations

AMR: Antimicrobial resistance
CSOs: Civil society organizations
GDF: Global Drug Facility
GHIT Fund: Global Health Innovation Technology Fund
GHP: Global Health Policy
iGHP: Institute for Global Health Policy Research
JATA: Japan Anti-Tuberculosis Association
JICA: Japan International Cooperation Agency
MDR-TB: Multi drug resistance – tuberculosis
MSF: Médecins Sans Frontières
NCGM: National Center for Global health and Medicine
NGOs: Non-governmental organizations
R&D: Research and Development
SDGs: Sustainable Development Goals
STBP: Stop-TB partnership
TB: Tuberculosis
UN: United Nation
WHO: World Health Organization

## References

[1] World Health Organization, Global Tuberculosis Report 2018. 2018. Available from http://apps.who.int/iris/bitstream/handle/10665/274453/9789241565646-eng.pdf?ua=1. Accessed 21 Nov 2018.

[2] STOP TB PARTNERSHIP & MEDECINS SANS FRONTIERES. Out of Step 2015 TB Policies in 24 countries, a survey of diagnostic and treatment practices. 2015. Available from http://www.stoptb.org/assets/documents/news/report_out_of_step_2015_11_pdf_with_interactive_links.pdf#search=%27Out+of+Step+2015+TB+Policies+in+24+Countries+About+Stop+TB+Partnership%27. Accessed 28 May 2018.

[3] World Health Organization. The End TB Strategy. 2015. Available from http://www.who.int/tb/End_TB_brochure.pdf?ua=1 .Accessed 28 May 2018.

[4] World Health Organization. Global Tuberculosis Report 2017: Leave no one behind - Unite to end TB. 2017. Available from http://www.who.int/tb/publications/global_report/gtbr2017_main_text.pdf?ua=1. Accessed 28 May 2018.

[5] Shawar YR, Shiffman J, Spiegel DA (2015). Generation of political priority for global surgery: a qualitative policy analysis. Lancet Glob Heal.

[6] Hafner T, Shiffman J (2013). The emergence of global attention to health systems strengthening. Health Policy Plan.

[7] Walt G, Shiffman J, Schneider H, Murray SF, Brugha R, Gilson L (2008). ‘Doing’ health policy analysis: methodological and conceptual reflections and challenges. Health Policy Plan.

[8] Shiffman J, Smith S (2007). Generation of political priority for global health initiatives: a framework and case study of maternal mortality. Lancet.

[9] Katsuda N, Hirosawa T, Reyer JA, Hamajima N (2015). Roles of public health centers (hokenjo) in tuberculosis control in Japan. Nagoya J Med Sci.

[10] Toru M (2002). Japan’s 100 years experience in TB trend and its countermeasures. J Japanese Soc Intern Med.

[11] The Global Fund. Drug-resistant tuberculosis. 2018. Available from https://www.theglobalfund.org/media/6651/publication_drug-resistanttuberculosis_focuson_en.pdf?u=636609567000000000. Accessed 28 May 2018.

[12] Kawatsu L, Uchimura K, Izumi K, Ohkado A, Ishikawa N (2016). Profile of tuberculosis among the foreign-born population in Japan, 2007--2014. West Pacific Surveill response J WPSAR.

[13] Japan Anti Tuberculois Association. 2008 Annual Report. 2008. Available from http://www.jata.or.jp/rit/ekigaku/en/statistics-of-tb/. Accessed 28 May 2018.

[14] European Centre for Disease Prevention and Control. Assessing the burden of key infectious diseases affecting migrant populations in the EU/EEA: technical report. 2014. Available from https://ecdc.europa.eu/sites/portal/files/media/en/publications/Publications/assessing-burden-disease-migrant-populations.pdf#search=%27Assessing+the+burden+of+key+infectious+diseases+affecting+migrant+populations+in+the+EU%2FEEA%27. Accessed 28 May 2018.

[15] World Health Organization. Roadmap for childhood tuberculosis: towards zero deaths. 2013. Available from http://apps.who.int/iris/bitstream/handle/10665/89506/9789241506137_eng.pdf?sequence=1/. Accessed 28 May 2018.

[16] World Health Organization. WHO treatment guidelines for drug-resistant tuberculosis: 2016 update. 2016. Available from: http://apps.who.int/iris/bitstream/10665/250125/1/9789241549639-eng.pdf?ua=1%0Ahttp://apps.who.int/iris/handle/10665/250125. Accessed 28 May 2018.

[17] Fuady A, Houweling TAJ, Mansyur M, Richardus JH (2018). Catastrophic total costs in tuberculosis-affected households and their determinants since Indonesia’s implementation of universal health coverage. Infect Dis poverty.

[18] Zhou C, Long Q, Chen J (2016). Factors that determine catastrophic expenditure for tuberculosis care: a patient survey in China. Infect Dis poverty..

[19] Getahun B, Wubie M, Dejenu G, Manyazewal T (2016). Tuberculosis care strategies and their economic consequences for patients: the missing link to end tuberculosis. Infect Dis poverty..

[20] Tanimura T, Jaramillo E, Weil D, Raviglione M, Lönnroth K (2014). Financial burden for tuberculosis patients in low- and middle-income countries: a systematic review. Eur Respir J.

[21] Moon S, Sridhar D, Pate MA (2015). Will Ebola change the game? Ten essential reforms before the next pandemic. The report of the Harvard-LSHTM independent panel on the global response to Ebola. Lancet.

[22] Daftary A, Calzavara L, Padayatchi N. The contrasting cultures of HIV and tuberculosis care. AIDS. 2015;29(1). 10.1097/QAD.0000000000000515 PMC4770012 25387315

[23] Courtwright A, Turner AN (2010). Tuberculosis and stigmatization: pathways and interventions. Public Health Rep.

[24] Ohmori M, Ishikawa N, Yoshiyama T, Uchimura K, Aoki M, Mori T (2002). Current epidemiological trend of tuberculosis in Japan. Int J Tuberc Lung Dis.

[25] Shimao T (2016). Peculiarity of national tuberculosis program. Japan Tuberc.

[26] Lönnroth K, Glaziou P, Weil D, Floyd K, Uplekar M, Raviglione M (2014). Beyond UHC: monitoring health and social protection coverage in the context of tuberculosis care and prevention. PLoS Med.

[27] Mauch V, Bonsu F, Gyapong M (2013). Free tuberculosis diagnosis and treatment are not enough: patient cost evidence from three continents. Int J Tuberc Lung Dis..

[28] Ukwaja KN, Modebe O, Igwenyi C, Alobu I (2012). The economic burden of tuberculosis care for patients and households in Africa: a systematic review. Int J Tuberc Lung Dis..

